# Children's and adolescents’ evaluations of wealth‐related STEM inequality

**DOI:** 10.1111/sode.12710

**Published:** 2023-09-22

**Authors:** Luke McGuire, Christina Marlow, Adam J. Hoffman, Angelina Joy, Fidelia Law, Adam Hartstone‐Rose, Adam Rutland, Mark Winterbottom, Frances Balkwill, Karen P. Burns, Laurence Butler, Grace Fields, Kelly Lynn Mulvey

**Affiliations:** ^1^ University of Exeter Exeter UK; ^2^ North Carolina State University Raleigh USA; ^3^ Cornell University Ithaca USA; ^4^ University of Cambridge Cambridge UK; ^5^ Centre of the Cell Queen Mary University of London London UK; ^6^ Virginia Aquarium & Marine Science Center Gloucester Point USA; ^7^ Birmingham Museums Trust Thinktank Science Museum Birmingham UK; ^8^ Riverbanks Zoo & Garden Columbia USA

**Keywords:** rectifying inequality, STEM inequality, wealth inequality

## Abstract

The fields of science, technology, engineering, and mathematics (STEM) are rife with inequalities and under‐representation that have their roots in childhood. While researchers have focused on gender and race/ethnicity as two key dimensions of inequality, less attention has been paid to wealth. To this end, and drawing from the Social Reasoning Development approach, we examined children's and adolescents’ perceptions of STEM ability and access to opportunities as a function of wealth, as well as their desire to rectify such inequalities. Participants (*n* = 234: early childhood, *n* = 70, mean age = 6.33, SD = .79; middle childhood, *n* = 92, mean age = 8.90, SD = .83 and early adolescence, *n* = 62, mean age = 12.00; SD = 1.16) in the U.K. (64% White British) and U.S. (40% White/European American) read about two characters, one high‐wealth and one low‐wealth. In early childhood, participants reported that the high‐wealth character would have greater STEM ability and were just as likely to invite either character to take part in a STEM opportunity. By middle childhood, participants were more likely to report equal STEM abilities for both characters and to seek to rectify inequalities by inviting the low‐wealth character to take part in a STEM opportunity. However, older participants reported that peers would still prefer to invite the high‐wealth character. These findings also varied by ethnic group status, with minority status participants rectifying inequalities at a younger age than majority status participants. Together these findings document that children are aware of STEM inequalities based on wealth and, with age, will increasingly seek to rectify these inequalities.

## 1 INTRODUCTION

Globally, wealth inequalities limit access to educational resources and contribute directly to educational outcomes (Aiyar & Ebeke, 2020; Pfeffer et al., 2018). Within science, technology, engineering, and mathematics (STEM), there are well‐documented gender‐ and race/ethnicity‐based disparities in education and workforce representation (Ma & Liu, 2017; Noonan et al., 2017; Riegle‐Crumb et al., 2019; WISE, 2017). Less well documented are disparities based on socio‐economic status (SES) or social class (Podobnik et al., 2020). This is important to recognise given the link between wealth inequality and educational outcomes, as well as the intersection between race/ethnicity and SES (Ma & Liu, 2017; Pfeffer et al., 2018). Since wealth inequalities continue to increase (Zucman, 2019), it is important to understand how youth perceive wealth inequalities in STEM contexts. Research has documented the perceptions of youth regarding the importance of rectifying inequality (Elenbaas et al., 2016; Rizzo & Killen, 2016, 2020; Rizzo et al., 2020) and their views on exclusion in the context of social class (Gönül et al., 2023). What are less well understood are whether youth make connections between wealth and STEM abilities, whether they perceive that there is inequitable access to STEM based on wealth, whether they seek to rectify inequalities in STEM contexts, and, how these judgments intersect with participant ethnic group status.

### 1.1 Wealth and science capital

Success in STEM is associated with *science capital* (i.e., knowledge, experiences and opportunities), however, some individuals have greater opportunity to engage with science, due to status, family affordances and cultural context (Archer et al., 2015). Science capital is unevenly distributed based on demographics like gender, race/ethnicity, and wealth (Archer et al., 2015; Podobnik et al., 2020). One area of uneven distribution is access to out‐of‐school STEM activities, such as at informal STEM learning sites (ISLS; e.g., museums or zoos), with visits to these sites associated with higher interest and abilities in STEM (Afterschool Alliance, 2014; Moore et al., 2014; Sahin et al., 2014; Social Mobility Commission, 2019). Given these links between inequalities in access to science capital and demographic variables, it is important to understand the beliefs that may motivate individuals to challenge and rectify such inequalities, for instance by including their peers who may not traditionally have access.

### 1.2 Theoretical framework

The current study utilized a Social Reasoning Developmental approach (SRD, Rutland & Killen, 2017; Rutland et al., 2010) which proposes that social judgments are made by considering both moral issues (e.g., “is this fair?”) and social domain issues like group norms (e.g., “what would others like me do in this situation?”; Rutland & Killen, 2017; Rutland et al., 2010). This model focuses on social judgments in general (especially those with morally relevant components) and has been applied to understanding youth's perceptions of wealth inequality (Killen et al., 2022). The SRD model also proposes that thinking about group processes leads to children considering the *status* of groups when making social decisions (Rutland & Killen, 2017). Consistent with an SRD perspective, the current study aims to understand how children and adolescents balance their understanding of *wealth* status with their intentions to include others in STEM activities and to make judgments about others’ STEM abilities. These judgments require coordination of information about societal structures, social expectations, moral principles around inclusion, and personal attitudes.

Since SRD emphasizes the importance of group norms and processes, it is important to consider not only the child's own judgments regarding inequality but also what they believe *other* group members would do in the situation. Perceptions of others’ beliefs play an important role in establishing group norms, which can have consequences for the likelihood of more inclusive decisions being made (Rizzo et al., 2018). Children typically believe that they themselves would resist a negative group norm, but that their in‐group members would continue to perpetuate biases (Killen et al., 2012; Mulvey & Killen, 2015). Therefore, depending on what children believe the group's norms are, their judgments may differ from what they believe the group will do. Recognising potential differences between the individual perspective and their perceived group perspective has important consequences for how children may behave, given the importance of peer group norms in guiding children's behaviour.

### 1.3 Children's knowledge of wealth inequality

Children as young as five‐to‐eight‐years‐old are sensitive to inequalities (Hazelbaker et al., 2018). They readily describe lower SES groups as having fewer resources, with children from lower SES groups being more likely to describe the struggle that accompanies this lack of resources than those in higher SES groups (Weinger, 2000). Children also attribute more negative attributes and less positive attributes toward those they view as poor compared to those they view as rich (Mistry et al., 2016). Views become more nuanced overtime with older adolescents being more likely to attribute poverty to societal or multidimensional factors compared to younger adolescents, who attribute poverty to more individualistic factors (Flanagan et al., 2014). However, adolescents, particularly those who are *not* disadvantaged, are often unaware of the true extent of wealth inequality and advocate for more egalitarian distributions than those seen in society (Arsenio & Willems, 2017).

Though children and adolescents may not always be aware of the extent of wealth inequalities, they do favour equality. Children as young as three years old attempt to rectify structural inequalities in luxury resources (Elenbaas et al., 2016; Rizzo & Killen, 2016, 2020; Rizzo et al., 2020). Children also attempt to rectify unequal access to opportunities, for example, when children aged eight to fourteen years were told about an informal educational opportunity (a zoo summer camp) that could only take on ten more children they preferred an equal representation of high and low‐wealth children (Elenbaas, 2019). However, when told that low‐wealth children had been previously excluded, participants were more likely to pick ten low‐wealth children for the zoo summer camp. In contrast, when told that high‐wealth children were previously excluded, participants were more likely to pick an equal distribution, rather than just picking the ten high‐wealth children (Elenbaas, 2019).

One possibility based on this literature is that children are aware that high‐wealth children have more opportunities than low‐wealth children regardless of prior exclusion in a single context. Thus, consistent with the SRD perspective (Rutland & Killen, 2015) and recent theorising about youth's understanding of social class (Mistry et al., 2021), there is evidence that children and young adolescents do take group *status* into account when making decisions about who to include in educational opportunities. However, more research is needed that explores how children make such inclusion decisions in STEM contexts, with attention to the role of wealth status and judgements of peers’ STEM abilities. For instance, children are less likely to rectify unequal distribution of STEM resources when the disadvantaged group is made up of girls and the advantaged group is made up of boys, demonstrating that STEM *stereotypes* influence judgments of fairness (Sims et al., 2022). Furthermore, recent research documents that adolescents make decisions about inclusion in STEM activities based on assumptions about ability, which are linked to individual characteristics like peers’ gender and race (Joy et al., 2023).

As previously discussed, SRD proposes that individuals make social decisions by considering elements of group identity and morality (Rutland et al., 2010). Therefore, it is important to consider how elements of group identity within a STEM context influence decisions regarding inclusion. Prior research has found that young children consider gender stereotypes when judging fairness of exclusion from STEM areas (Mulvey & Irvin, 2018). Furthermore, adolescents were more likely to include a non‐White peer in a STEM context if they felt that others in their school were less likely to endorse ethnic stereotype (Joy et al., 2023). Together, these studies suggest that children and adolescents take both their own STEM stereotypes *and* their perceptions of the stereotypes that their peers hold into consideration when determining who to include in a STEM context. The current study aimed to extend this work by examining wealth‐status ability stereotypes.

### 1.4 Race/ethnicity and wealth status

Race/ethnicity is an important characteristic of group membership that intersects with wealth status (Ma & Liu, 2017). Between 3‐ and 11‐years‐old, children increasingly understand with age that rich peers have greater access to resources, and this increased access is especially true for White peers compared to Black peers (Elenbaas et al., 2022). This understanding influences social decision making. For example, one study asked 8–14‐year‐old U.S. children to make predictions about how inclusive high‐wealth and low‐wealth peers would be, as well as how inclusive African American and European American peers would be. Older children predicted that high‐wealth peers would be less inclusive, irrespective of the race of the peers (Burkholder et al., 2020). Further, research has shown that European American and African American children use different criteria when deciding who to include in an after‐school club. Specifically, European American children expected their peers to choose a same‐wealth peer even if they were a different race, whereas African American children expected their peers to choose a same‐race peer even if they were a different level of wealth (Burkholder et al., 2021). These findings suggest that in middle childhood there is an emerging understanding of the intersection of wealth and race/ethnicity, which informs peer inclusion decisions. To complement our central focus on children's understanding of the influence of wealth status in STEM, we also examined how ethnic majority or minority status related to judgments about STEM ability and inclusion decisions.

### 1.5 Current study

The present study aimed to investigate (1) how wealth is related to children's and adolescents’ judgments about inclusion of peers in STEM opportunities, (2) whether there are specific wealth‐based stereotypes regarding STEM ability and (3) whether children believed they and their peers would attempt to rectify or perpetuate these inequalities. First, we hypothesized (H1) that children would attribute greater STEM ability to high‐wealth children than low‐wealth children, however we had no specific hypotheses regarding age effects. Consistent with an SRD perspective, we expected that moral principles may take precedence over ability judgements: we hypothesized (H2) that based on children's existing knowledge of inequality, children would be more likely to include a low‐wealth child than a high‐wealth child and that this would increase with age. Additionally, we expected (H3) that in a peer selection task (i.e., choosing who ought to take the final place on a trip to a science museum) children and adolescents would believe that others would be less likely to include a low‐wealth peer than they would individually. This was expected due to norms regarding access and ability and that this belief would increase with age. Finally, we examined the role of participants’ own ethnic group status (minority, majority) as an exploratory factor in the preceding analyses, with no directional hypotheses.

## 2 METHOD

### 2.1 Participants

#### 2.1.1 National contexts

Data were collected in the U.K. and the U.S., where there are similarities in wealth inequalities and STEM disparities. The Gini index is a measure of wealth inequality ranging from 0 (total equality, e.g., wealth is evenly distributed between all individuals in a nation) to 100 (total inequality, e.g., all wealth in a nation is held by one person). In 2020, the U.K. had a Gini index of 32.6, while the U.S. had a Gini index of 39.7. Representation of women is also comparable in the U.K. and U.S.; around 24% of U.K. individuals working in STEM are women (STEM Women, 2022) and around 34% of U.S. individuals working in STEM are women (National Science Board, 2021). While the racial/ethnic groups that are considered to be minoritized may differ by country, ratio of representation compared to the whole population demographics in STEM fields is fairly comparable between the two nations. In the U.K. the vast majority (87%) of workers in STEM identify as White (All‐Party Parliamentary Group on Diversity and Inclusion in Science & Engineering and Maths, 2021) and in the U.S. the majority (65%) also identify as White (National Science Board, 2021). STEM inequalities between the two nations on the basis of SES or social class are not well documented, although some studies reflect that in both the U.S. and U.K. children and adolescents who are low SES predict greater barriers to their involvement in and demonstrate less interest in STEM (Codiroli Mcmaster, 2017; Turner et al., 2017).

### 2.2 Sample

Power analysis using G*Power (Faul et al., 2007) suggested that to observe a small effect size (*d* = .25) with power of .80, within the design of our task (age measured at three levels, gender at two levels, ethnic group status at two levels) we would require 158 participants. A total of 234 participants were recruited from four ISLS in the U.K. and U.S.

In the U.K., participants (female *n* = 66, male *n* = 70, didn't report gender *n* = 4) were recruited from a science museum in the Midlands (area including major cities Birmingham, Coventry, Nottingham; *n* = 104) and a science centre in the South‐East (area including major cities London, Brighton, Canterbury; *n* = 36). For the purposes of analysis, participants were divided into three age groups: early childhood (*n* = 32, 5–7‐years‐old, mean age = 6.53, *SD* = 0.62), middle childhood (*n* = 59, 8–10‐years‐old, mean age = 8.81, *SD* = 0.84) and early adolescence (*n* = 40, 11–16‐years‐old, mean age = 11.83, *SD* = 1.06). In both the U.K. and U.S. participants were asked to self‐report their race/ethnicity, but if they were unable to do so the experimenter would ask the child's parent for confirmation. The race/ethnicity of participants in the U.K. sample was as follows: 89 participants (64%) reported their race/ethnicity as White British, 18 (11%) as South Asian British (including Bengali, Indian & Pakistani), 10 (7%) as Mixed Race or Dual Heritage, three (2%) as Black British, three (2%) as Chinese British, six (4%) as ‘other’ than those categories we provided, and 11 (8%) participants did not report their race/ethnicity.

In the U.S., participants (female *n* = 52, male *n* = 39, didn't report gender *n* = 3) were recruited from an aquarium (*n* = 84) and a zoo (*n* = 10), both in the Southeastern U.S (area including states North Carolina, South Carolina, Tennessee). Again, participants were divided into three age groups: early childhood (*n* = 38, 4–7‐years‐old, mean age = 6.16, *SD* = 0.89), middle childhood (*n* = 33, 8–10‐years‐old, mean age = 9.06, *SD* = 0.79) and early adolescence (*n* = 22, 11–16‐years‐old, mean age = 12.32, *SD* = 1.29). The race/ethnicity of participants in the U.S. sample was as follows: 38 (40%) participants reported their race/ethnicity as White/European American, six (6%) as Bi‐racial or Multi‐racial, six (6%) as Black/African American, two (2%) as Hispanic/Latino, one (1%) as Asian/Asian American, four (4%) as ‘other’ than those categories we provided; 37 (39%) participants did not report their race/ethnicity.

Due to the pragmatic restrictions of collecting data in these institutional settings, we were not able to measure the SES of our participants. On average, data suggests that individuals from the most deprived areas (based on the Index of Multiple Deprivation, calculated using a variety of measures including income deprivation, crime and living environment deprivation) in the U.K. are less likely to have visited museums or galleries compared to those from the least deprived areas (34% from most deprived areas, 59% from least deprived areas; Department for Digital, Culture, Media & Sport, 2020). In the U.S., one estimate of the average income of science museum visitors in 2018 was between $57,000 and $59,000 which is above the poverty line (Dilenschneider, 2018) but comparable to the median income of $61,937 in the U.S. according to the 2018 US Census (U.S. Census Bureau, 2019). Furthermore, the majority of children who visit museums in the U.S. have parents who obtained at least some education post‐high school level, another indicator of SES, and visitors tend to be White or European American (Gold, 2021).

### 2.3 Procedure

All measures were approved by the North Carolina State University IRB as part of the STEM Teens project in the U.S. and the ethics committee of Goldsmiths, University of London in the U.K. The protocol was completed using online survey software (Qualtrics, Provo, UT) on a tablet computer, or in hard copy. In both cases the same measures were used. Participants could complete the survey independently or in a one‐to‐one interview format with an experimenter if they were not confident in their reading ability. Parental consent and child assent were obtained for all participants in the U.K. and parental notification and child assent established for all participants in the U.S.

Participants were recruited from family groups visiting the site, containing at least one adult and one child per group, and offered either an electronic gift card, gift shop voucher or gift bag worth £/$5 for completing a questionnaire. All participants were approached at the exit of pre‐selected galleries or exhibitions. The measures presented below were part of a larger questionnaire that included measures related to STEM ethnic stereotypes, STEM self‐efficacy and learning motivation.

### 2.4 Materials

Participants were first asked to imagine a character called Alex (high‐wealth character). They were told that “Alex lives in this house, takes this backpack to school, and their parents drive this car”. This text was accompanied by pictures of a large house, modern car, and new backpack. Appropriate cars and houses for the U.S. and U.K. contexts were used. These items have been used as reliable indicators of wealth status in studies with children in this age range (Elenbaas, 2019). No image of Alex was provided and gender‐neutral pronouns were used, so the gender and race/ethnicity of this and the low‐wealth character were not specified..

**STEM ability**: Participants were asked: “how good do you think Alex would be at science?” (1 = *really not good*, 6 = *really good*) and “how good do you think Alex would be at math(s)?” (1 = *really not good*, 6 = *really good*).

**ISLS access**: Next, participants were asked “how often do you think Alex goes to science centers, zoos and aquariums?” (1 = *never*, 5 = *once a week*).

This procedure was repeated for the low‐wealth character, Sam. Sam's gender and race/ethnicity were also not specified. Pictures of Sam's smaller house, older backpack and older car were provided. The same questions about Sam's science and math ability, and ISLS access were asked.

Participants read the following hypothetical scenario: “Alex and Sam both go to the same school. The school has a new after school science club where some kids will get to work on fun science projects and visit science centers to learn more. Here are some kids who are part of the after‐school science club.” This was accompanied by a picture of six silhouettes of children including boys and girls. Participants then read “both Alex and Sam would like to join the after‐school science club, who are going on a trip to the local science museum. There is only room for one more person to join the club for this trip.”

**Peer selection**: Participants were asked “who would you invite to join the club for this trip?” (binary choice between high and low‐wealth characters) and “why would you invite this person?” (open‐ended response).

**Perceived group choice**: Participants were asked “who do you think the rest of the club would invite to join them for this trip?” (binary choice between high and low‐wealth characters) and “why do you think the club would invite that person?” (open‐ended response).

### 2.5 Data analytic plan

To account for the multi‐site nested nature of our data we calculated intra‐class correlation coefficients (ICCs) as a function of site to assess whether multi‐level modelling was appropriate. Given the low ICCs for STEM ability (.013) and ISLS access (.01) questions by site it was determined that multi‐level modelling was not necessary.

The math and science ability questions for both the high‐wealth (*r* = .57, *p* = .001) and low‐wealth (*r* = .53, *p* = .001) characters were positively correlated. Therefore, we created a composite STEM ability score for each character by averaging across these two questions. We carried out repeated measures ANOVAs to assess differences in the repeated measures STEM ability score (high‐wealth character, low‐wealth character) as a function of participant age and ethnic group status. For the purposes of exploratory analysis, we compare majority status participants (White British, European American) and minority status participants (all other ethnic groups). Participants who did not provide their race/ethnicity were excluded from these analyses. We included gender in these models to control for this variable. In the analyses reported below there were no effects of gender and hence it is not mentioned further. Pairwise comparisons were carried out with Bonferroni corrections for multiple comparisons applied. The same repeated measures ANOVA approach was used to assess differences in the ISLS access question. Chi‐square analysis was used to assess differences in peer selection and perceived group choice questions to assess whether choice of high‐wealth versus low‐wealth character differed as a function of age group and ethnic group status.

To assess the open‐ended justifications for both individual peer selection and perceived group peer selection, a coding framework (see Table 1) was developed drawing from a reading of the data and existing literature in the area (Elenbaas, 2019). Two coders, blind to the hypotheses of the study, conducted the coding. Inter‐rater reliability procedures indicated strong agreement between the coders for both the individual response reasoning (Cohen's *κ* = .98) and perceived group response reasoning (Cohen's *κ* = .97). To follow up on our analyses of peer selection, we use layered chi‐square tests to assess whether there are differences in participants’ use of coding categories based on their age group and chosen peer.

**TABLE 1:** Reasoning category framework.

Category	Definition	Examples
1. Rectify Inequality	Any reference to choosing the poor character because they do not have as much access to ISLS	“Alex looks like he has money and goes more often” “They have less opportunities to do things like this”
2. Wealth Status	Any reference (positive or negative) to the wealth status of the individual or their parents/family	“Better book bag and nicer car” “Because he has a nicer car and house”
3. Personal Characteristics	Any reference to personality traits or popularity of the character (not explicitly related to their wealth status)	“Because he's kind” “He's popular”
4. Intelligence/STEM Ability	Any reference to the intelligence or STEM‐related abilities of the character	“Because I think he knows a lot more information than Sam” “I think he is better at Science”

## 3 RESULTS

### 3.1 STEM ability

**FIGURE 1:**
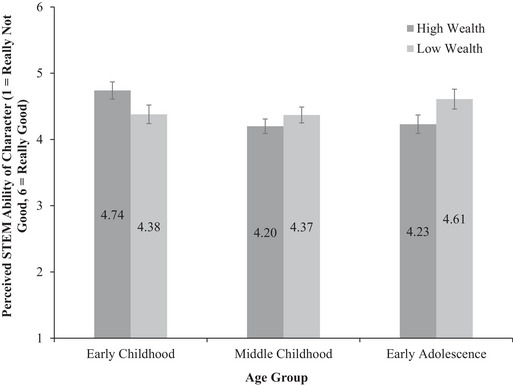
Evaluations of STEM ability of high‐wealth and low‐wealth characters as a function of participant age group (with standard error bars, *n* = 179; early childhood 5–7‐years‐old, *n* = 50, middle childhood 8–10‐years‐old, *n* = 75, early adolescence, 11–16‐years‐old, *n* = 54).

Testing H1, within‐subjects effects revealed a significant interaction between participant age group and our repeated measures STEM ability variable, *F*(2, 172) = 3.75, *p* = .03, *η_p_* ^2^ = .04 (see Figure 1). Participants in early childhood rated the high‐wealth character (*M* = 4.74, *SD* = 1.25) as having greater STEM ability than the low‐wealth character (*M* = 4.38, *SD* = 1.23, *p* = .02). In middle childhood there was no difference between ratings of the ability of the high‐wealth character (*M* = 4.20, *SD* = 1.06) and the low‐wealth character (*M* = 4.37, *SD* = 1.13, *p* = .91). In the early adolescence group there was no difference in ratings of the ability of the low‐wealth character (*M* = 4.61, *SD* = .98) and the high‐wealth character (*M* = 4.23, *SD* = .99, *p* = .15).

**FIGURE 2:**
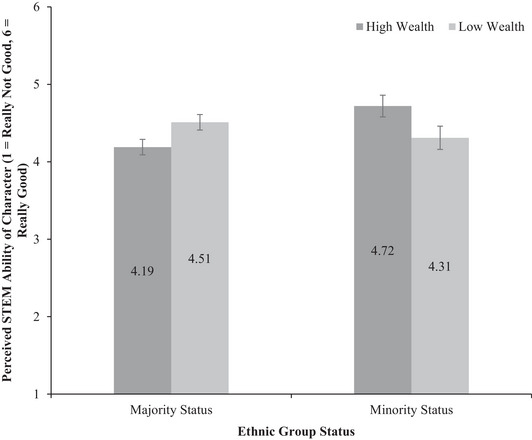
Evaluations of STEM ability of High‐wealth and Low‐wealth characters as a function of participant ethnic group status (with standard error bars, *n* = 179, majority status (incl. White British & White/European American participants) *n* = 122, minority status (incl. all other ethnic groups) *n* = 57).

We also observed a significant two‐way interaction between ethnic group status and our repeated measures STEM ability variable, *F*(1, 172) = 9.27, *p* = .003, *η_p_* ^2^ = .05 (see Figure 2). Minority status participants reported that the high‐wealth character (*M* = 4.72, *SD* = 1.03) had higher STEM ability than the low‐wealth character (*M* = 4.31, *SD* = 1.04, *p* = .03). In contrast, majority status participants reported that the low‐wealth character (*M* = 4.51, *SD* = 1.15) had higher STEM ability than the high‐wealth character (*M* = 4.19, *SD* = 1.12, *p* = .03).

### 3.2 ISLS access

Testing H2, within‐subjects effects revealed a main effect of character wealth on our repeated measures ISLS access variable, *F*(1, 173) = 6.28, *p* = .01, *η_p_* ^2^ = .04. Participants perceived that the high‐wealth character (*M* = 3.23, *SD* = 1.29) had greater access to ISLS than the low‐wealth character (*M* = 2.72, *SD* = 1.27). There was no interaction between the repeated measures ISLS access variable and participant age group, or with ethnic group status.

### 3.3 Peer selection

Testing H3, chi‐square analysis suggested that participants’ peer selection decisions differed based on their age group, *χ* ^2^ (2) = 25.43, *p* < .001, Cramer's *V* = .27 (see Table 2). Fifty‐three percent of participants in early childhood selected the high‐wealth character (*n* = 27) and 47% selected the low‐wealth character (*n* = 24). In the middle childhood group, 76% of participants selected the low‐wealth character (*n* = 57), compared to 24% who selected the high‐wealth character (*n* = 18). In the early adolescence group, 91% of participants selected the low‐wealth character (*n* = 48), while 9% selected the high‐wealth character (*n* = 5).

**TABLE 2:** Character choice as a function of age and ethnic group status in individual choice and perceived peer choice tasks.

Ethnic group status	Age group	Task	High‐wealth character	Low‐wealth character
Majority	Early childhood	Individual choice	20 (59%)	14 (41%)
		Perceived peer choice	21 (66%)	11 (34%)
	Middle childhood	Individual choice	17 (31%)	37 (69%)
		Perceived peer choice	44 (83%)	9 (17%)
	Early adolescence	Individual choice	4 (11%)	33 (89%)
		Perceived peer choice	25 (69%)	11 (31%)
Minority	Early childhood	Individual choice	7 (41%)	10 (59%)
		Perceived peer choice	11 (65%)	6 (35%)
	Middle childhood	Individual choice	1 (5%)	20 (95%)
		Perceived peer choice	20 (91%)	2 (9%)
	Early adolescence	Individual choice	1 (6%)	15 (94%)
		Perceived peer choice	10 (63%)	6 (37%)

These effects were qualified by the addition of ethnic group status as a layer in the chi‐square analysis. For minority status participants (*χ* ^2^ (2) = 10.75, *p* = .006, Cramer's *V* = .32), in early childhood 59% of participants (*n* = 10) selected the low‐wealth character, while 41% (*n* = 7) selected the high‐wealth character. In middle childhood, 95% (*n* = 20) selected the low‐wealth character, while 5% (*n* = 1) selected the high‐wealth character. In early adolescence, 94% (*n* = 15) selected the low‐wealth character, while 6% (*n* = 1) selected the high‐wealth character).

For majority status participants (*χ* ^2^ (2) = 18.61, *p* < .001, Cramer's *V* = .27), in early childhood 59% (*n* = 20) selected the high‐wealth character, while 41% (*n* = 14) selected the low‐wealth character. In middle childhood, 69% (*n* = 37) selected the low‐wealth character, while 32% (*n* = 17) selected the high‐wealth character. Finally, in early adolescence, 89% (*n* = 33) selected the low‐wealth character, while 11% (*n* = 4) selected the high‐wealth character.

### 3.4 Peer selection reasoning

Layered chi‐square analysis suggested that participants’ use of reasoning categories, to justify why they would select their chosen peer, differed based on their age group and whether participants selected the low‐wealth or high‐wealth peer (see Table 3). The same age‐related pattern of results was evident for majority and minority status participants.

**TABLE 3:** Reasoning category use for individual character selection task as a function of character choice and participant age group.

Character choice	Reasoning code	Early childhood	Middle childhood	Early adolescence
High‐wealth character	Rectify inequality	1 (4%)	1 (7%)	0 (0%)
	Wealth status	13 (52%)	6 (43%)	1 (25%)
	Personal characteristics	4 (16%)	1 (7%)	1 (25%)
	Intelligence/STEM ability	7 (28%)	6 (43%)	2 (50%)
Low‐wealth character	Rectify inequality	20 (74%)	40 (67%)	31 (78%)
	Wealth status	4 (15%)	5 (8%)	2 (5%)
	Personal characteristics	0 (0%)	5 (8%)	4 (10%)
	Intelligence/STEM ability	3 (11%)	10 (17%)	3 (8%)

For those in early childhood (*χ* ^2^ (3) = 27.52, *p* < .001, Cramer's *V* = .40), participants who selected the low‐wealth character were more likely to reference rectifying inequality than those who selected the high‐wealth character. In contrast, those who selected the high‐wealth character were more likely to reference the wealth of this character as a positive trait, or to reference positive personal characteristics of the peer.

A similar pattern for use of the rectifying inequality code when selecting the low‐wealth peer and referencing wealth when selecting the high‐wealth peer, occurred in middle childhood (*χ* ^2^ (3) = 19.98, *p* < .001, Cramer's *V* = .34). Further, in middle childhood those who selected the low‐wealth peer were more likely to reference intelligence or STEM specifically.

In early adolescence (*χ* ^2^ (3) = 11.73, *p* = .008, Cramer's *V* = .26), participants who selected the low‐wealth peer made greater reference to rectifying inequality than peers who selected the high‐wealth peer and made references to the intelligence or STEM ability of the low‐wealth character.

Looking for age effects within participants who selected the low‐wealth peer (*χ* ^2^ (6) = 2.74, *p* = .84, Cramer's *V* = .13) or high‐wealth peer (*χ* ^2^ (6) = 6.41, *p* = .38, Cramer's *V* = .19) did not reveal significant differences based on participant age. Looking across both peers (*χ* ^2^ (6) = 15.59, *p* = .02, Cramer's *V* = .30), there was a difference between early childhood, middle childhood, and early adolescence with a greater proportion of references to rectifying inequality in the older age groups. There were also greater references to wealth status in early childhood compared to those in middle childhood or adolescence.

### 3.5 Perceived group choice

Chi‐square analysis suggested that participants’ perceptions of who the rest of their group would choose to include differed based on their age group, *χ* ^2^ (2) = 8.22, *p* = .02, Cramer's *V* = .15 (see Table 2). In early childhood, 65% of participants expected their peers to select the high‐wealth character (*n* = 32), and 35% expected their peers to select the low‐wealth character (*n* = 17). In middle childhood, 85% of participants expected their peers to select the high‐wealth character (*n* = 64), while 15% expected their peers to select the low‐wealth character (*n* = 11). Finally, in early adolescence, 67% of participants expected their peers to select the high‐wealth character (*n* = 35), while 33% expected their peers to select the low‐wealth character (*n* = 17). These effects did not differ as a function of ethnic group status.

### 3.6 Perceived group choice reasoning

Layered chi‐square analysis suggested that participants’ use of reasoning categories (see Table 4), to justify why they thought the other members of the peer group would select their chosen character, did not differ in early childhood (*χ* ^2^ (3) = 4.18, *p* = .24, Cramer's *V* = .16). In middle childhood (*χ* ^2^ (3) = 30.85, *p* < .001, Cramer's *V* = .44), participants who believed their peers would select the low‐wealth character were more likely to reference rectifying inequality. In contrast, those who believed their peers would select the high‐wealth character were more likely to reference the wealth status of the character as a positive. In early adolescence (*χ* ^2^ (3) = 18.21, *p* < .001, Cramer's *V* = .34), the same pattern was apparent. The same age‐related pattern of results was evident for majority and minority status participants.

**TABLE 4:** Reasoning category use for perceived peer character selection task as a function of perceived peer character choice and participant age group.

Character choice	Reasoning code	Early childhood	Middle childhood	Early adolescence
High‐wealth character	Rectify inequality	2 (7%)	1 (2%)	0 (0%)
	Wealth status	15 (48%)	36 (56%)	15 (50%)
	Personal characteristics	4 (13%)	17 (27%)	11 (37%)
	Intelligence/STEM ability	10 (32%)	10 (16%)	4 (13%)
Low‐wealth character	Rectify inequality	3 (21%)	6 (46%)	4 (44%)
	Wealth status	3 (21%)	1 (8%)	0 (0%)
	Personal characteristics	3 (21%)	2 (15%)	3 (33%)
	Intelligence/STEM ability	5 (36%)	4 (31%)	2 (22%)

Looking for age effects within participants who selected the high‐wealth character (*χ* ^2^ (6) = 10.46, *p* = .11, Cramer's *V* = .25) or low‐wealth character (*χ* ^2^ (6) = 4.95, *p* = .55, Cramer's *V* = .18) did not reveal significant differences based on participant age. Nor were there differences looking at all participants across both peer selection choices (*χ* ^2^ (6) = 8.28, *p* = .22, Cramer's *V* = .23).

## 4 DISCUSSION

Prior research has found that children become increasingly aware of wealth inequality and try to rectify it through inclusion of low‐wealth peers (Elenbaas, 2019; Hazelbaker et al., 2018). However, it is unknown whether and how STEM stereotypes regarding wealth may affect children's and adolescents’ judgments of inclusion and ability in situations involving wealth inequality in STEM contexts. The current study revealed that older participants were more likely to believe that a high‐wealth individual would have greater access to ISLS, but that they wouldn't necessarily have stronger STEM abilities. In contrast, younger children were more likely to say that a high‐wealth individual would have stronger STEM abilities than a low‐wealth individual. Crucially, with age, participants were more likely to choose a low‐wealth individual for a STEM opportunity due to wanting to rectify wealth‐based inequalities in STEM access. Ethnic minority status participants rectified this inequality at an earlier age than their majority status peers.

### 4.1 Perceived STEM ability & access

In line with prior work that found that late‐elementary aged children tend to attribute more positive characteristics such as academic ability to high‐wealth individuals as opposed to low‐wealth individuals (Mistry et al., 2016), young children rated the high‐wealth character as having greater STEM ability than the low‐wealth character. In contrast, however, children in middle childhood and early adolescence rated both individuals as similarly capable. These findings suggest that there are early wealth‐based stereotypes regarding STEM ability. Furthermore, in middle childhood and adolescence, participants also stated that the high‐wealth character would have greater access to ISLS than would the low‐wealth character. These findings, taken together, are in line with the prior literature that demonstrates a growing awareness in middle childhood and early adolescence of wealth disparity and the related resource and opportunity disparity (Arsenio & Willems, 2017; Hazelbaker et al., 2018).

The important distinction here lies in the finding that older children and adolescents understand that even though a high‐wealth child may have greater access to STEM opportunities or science capital, this does not necessarily relate to greater ability. This finding aligns with similar work on gender, that suggests in engineering and technology especially, there is an early tendency to suggest boys will have greater ability than girls, before later reporting that gender groups will have equal abilities (McGuire et al., 2022). Understanding the early emergence of attitudes that favour high‐wealth peers will be an important next step for research in this area. Within the bounds of the SRD model, this may speak to the early importance of status, which younger children associate with ability. With age, participants’ own experiences in the classroom may inform their understanding that wealth is not necessarily a prerequisite for success, even if it does afford opportunity.

### 4.2 Rectifying inequality

In line with their growing awareness about the disparity in access between high and low‐wealth individuals, most participants in middle childhood and early adolescence selected the low‐wealth character over the high‐wealth character as their choice for joining the trip. Furthermore, when asked why they chose the low‐wealth character, they directly referenced the prior inequality, implying a want to rectify the situation by explicitly including peers who may have been previously excluded because of wealth. Children and adolescents of these ages are able and ready to have discussions about wealth‐based inequality, and interventions should focus on empowering these individuals to feel they can do something about such inequalities.

Among our participants in early childhood, the pattern of results differed based on the ethnic group status of the participant. Young children from ethnic minority groups had a tendency to select the low‐wealth peer and rectify the inequality, while young children from ethnic majority groups demonstrated a tendency to choose the high‐wealth character, thus perpetuating the inequality. This finding speaks to the intersectional nature of wealth and ethnic group status; early socialization as a member of a minoritized group may result in greater awareness of systemic inequalities (Rosette & Tost, 2013) and greater likelihood of seeking to rectify such inequalities. In contrast, ethnic majority group members are less likely to experience such inequalities from a young age and caregivers are less likely to discuss such issues with their children (Priest et al., 2014). This finding opens the door to further work to probe this interesting initial effect with a crucial need for replications using stratified sampling that can go beyond a simple majority/minority status binary distinction.

In terms of reasoning, even young children were likely to reference resolving inequality if they chose the low‐wealth character and to reference wealth as a positive attribute if they chose the high‐wealth character. There appear to be two competing factors that may shape rectifying decisions: if the high‐wealth peer was chosen, participants used wealth ability stereotypes, while if the low‐wealth peer was chosen, participants referenced fairness principles. There appears to be a shift with age in which factor supersedes the other when making inclusion decisions. It may not be that young children are simply disregarding the wealth inequality or wealth stereotypes in favour of equality; more so that it is equally likely for young children to either endorse wealth‐based stereotypes or to begin to be aware of wealth‐based disparity in access to STEM opportunities and wish to rectify those.

Future research should continue to explore what predicts this shift towards emphasizing fairness principles over wealth‐based ability norms that are likely rooted in stereotypes. For instance, research might explore the role of social‐cognitive abilities such as theory of mind. These findings demonstrate that even young children can be aware of wealth‐disparities and utilize that information to inform decisions about access to STEM opportunities. Future research should continue to probe when in early childhood this awareness and desire to rectify wealth‐based inequality begins to occur and why some younger children may be more likely to wish to rectify the wealth‐based inequality than others.

### 4.3 Individual versus perceived peer decisions

Although most adolescents and middle‐childhood participants said they would include the previously disadvantaged low‐wealth character, increasingly with age, most participants thought that their peers would choose the high‐wealth character over the low‐wealth character. This finding is in line with prior research from the SRD perspective demonstrating that children believe they would resist negative norms, such as perpetuating inequality, but that groups will continue to promote inequality if it is the group norm (Killen et al., 2012; Mulvey & Killen, 2015). This underlies the importance of making adolescents aware of how most of their peers disagree with these negative inequality norms. Accurate perceptions of peers’ desire to rectify inequality may help encourage individuals to intervene as the fear of rejection, a common reason for not intervening, could decrease (Bennett et al., 2014). Open communication about negative social norms may lead to more explicit support of low‐wealth peers in STEM contexts, helping to diminish disparities seen in STEM fields, particularly given that students who leave STEM often cite feeling a lack of belonging (Rainey et al., 2018).

### 4.4 Limitations and future directions

Although the evidence is in line with prior research and extends findings on youth's understanding of wealth inequality to the STEM realm, there are some limitations to the current study. The cross‐sectional nature of the study may mean that age‐based differences may be in part due to cohort effects rather than only developmental change. Future studies should use longitudinal methods to track children's reasoning about wealth‐based inequalities to better understand what elements are developmentally driven. The participants in this study were, at the time, visiting ISLS. It is possible these children may be more aware of STEM based inequalities due to their own experiences in accessing an ISLS or could have been inadvertently primed to be thinking about access to STEM due to their surroundings. Therefore, future studies should explore whether the trends found, regarding STEM ability and access, continue to hold when children are not in a STEM relevant space. While the data collection context provides an important caveat, it is also worth considering as a positive for practitioners in this area. One possibility is that visiting ISLS and informal STEM settings that have more inclusive environments and representative staff can impact participants’ own attempts to rectify inequalities in this area. Indeed, researchers have shown that interactions with diverse staff in STEM settings is related to interest and motivation (McGuire et al., 2021). This will be a fruitful area for future research when comparing between formal and informal STEM contexts.

While we included participant ethnic group status in our models, group status was treated as a binary of majority or minority group which compared the White British and White European American participants against all other ethnic groups. This was necessary to ensure that we had the statistical power needed to assess differences between groups but does not reflect the different lived experiences of the many groups represented by our ‘minority’ designation. Future work should use a stratified sampling approach to replicate and extend the current findings and we urge caution in interpreting our exploratory findings around more nuanced ethnic group status. In addition, it is important to note that 40% of participants in the US did not report their race/ethnicity. Again, we urge caution in interpreting the findings of this exploratory analysis as a result of this and stress the importance of future work in this area that stratifies the sample by race/ethnicity.

Additionally, our task represented participants with images of their house, car, and backpack, but we did not include an image of the high‐wealth or low‐wealth target. This was to remove the influence of the target's race/ethnicity or gender. It would be interesting for future research to explicitly examine this influence, in particular examining children's perceptions of the intersection of race/ethnicity and wealth to observe whether their estimates of ability or rectifying depend on both the wealth and race/ethnicity of the target.

Finally, due to pragmatic restrictions of collecting data in the ISLS setting, we were unable to collect participant SES information, as we did not expect children to be able to accurately report their own familial SES. As the participants’ SES‐related lived experiences almost certainly influenced thinking about the tested scenarios, it will be essential to replicate our findings while explicitly examining the effect of participant SES as a valuable next step in this line of enquiry.

## 5 CONCLUSION

Overall, our findings extend the prior literature regarding children's and adolescents’ awareness of wealth‐based inequality and beliefs about rectifying these inequalities to a STEM context. Young ethnic majority children are less likely to rectify prior access to STEM opportunities, but as children age into middle childhood and adolescence, they become increasingly aware of wealth‐based STEM inequalities and wish to rectify them, even though they do believe they are a norm others would choose to perpetuate. Our findings demonstrate that children and adolescents are aware of wealth‐based inequalities and therefore should be involved, potentially through direct interventions, in helping to rectify individual‐level wealth‐based disparities. As children age, they do wish to rectify the wealth‐based inequalities they see in the world, regardless of whether they believe others feel the same way as them.

## CONFLICT OF INTEREST STATEMENT

The authors declare they have no relevant financial or non‐financial interests to disclose.

## Data Availability

The author has provided the required Data Availability Statement, and if applicable, included functional and accurate links to said data therein.
